# A Mutation in *DAOA* Modifies the Age of Onset in *PSEN1* E280A Alzheimer's Disease

**DOI:** 10.1155/2016/9760314

**Published:** 2016-01-05

**Authors:** Jorge I. Vélez, Dora Rivera, Claudio A. Mastronardi, Hardip R. Patel, Carlos Tobón, Andrés Villegas, Yeping Cai, Simon Easteal, Francisco Lopera, Mauricio Arcos-Burgos

**Affiliations:** ^1^The Arcos-Burgos Group, Department of Genome Sciences, John Curtin School of Medical Research, The Australian National University, Canberra, ACT 2600, Australia; ^2^Neuroscience Research Group, University of Antioquia, Medellín, Colombia; ^3^The Easteal Group, Department of Genome Sciences, John Curtin School of Medical Research, The Australian National University, Canberra, ACT 2600, Australia

## Abstract

We previously reported age of onset (AOO) modifier genes in the world's largest pedigree segregating early-onset Alzheimer's disease (AD), caused by the p.Glu280Ala (E280A) mutation in the *PSEN1* gene. Here we report the results of a targeted analysis of functional exonic variants in those AOO modifier genes in sixty individuals with *PSEN1* E280A AD who were whole-exome genotyped for ~250,000 variants. Standard quality control, filtering, and annotation for functional variants were applied, and common functional variants located in those previously reported as AOO modifier loci were selected. Multiloci linear mixed-effects models were used to test the association between these variants and AOO. An exonic missense mutation in the *G72* (*DAOA*) gene (rs2391191, *P* = 1.94 × 10^−4^, *P*_FDR_ = 9.34 × 10^−3^) was found to modify AOO in *PSEN1* E280A AD. Nominal associations of missense mutations in the *CLUAP1* (rs9790, *P* = 7.63 × 10^−3^, *P*_FDR_ = 0.1832) and *EXOC2* (rs17136239, *P* = 0.0325, *P*_FDR_ = 0.391) genes were also found. Previous studies have linked polymorphisms in the *DAOA* gene with the occurrence of neuropsychiatric symptoms such as depression, apathy, aggression, delusions, hallucinations, and psychosis in AD. Our findings strongly suggest that this new conspicuous functional AOO modifier within the *G72* (*DAOA*) gene could be pivotal for understanding the genetic basis of AD.

## 1. Introduction

Alzheimer's disease (AD, OMIM 104300), the most common type of dementia, is a neurodegenerative disorder characterized by learning disabilities, cognitive decline, aggression, and short- and long-term memory loss [1]. Mutations in the* Presenilin-1* (*PSEN1*) [2],* Presenilin-2 *(*PSEN2*) [3], and* amyloid precursor protein *(*APP*) [4] genes cause early-onset AD (EAOD). A rare mutation (with a minor allele frequency [MAF] of <1%) in* APP* had a protective effect against AD in Icelanders [5], whilst a rare mutation in the* Phospholipase D family member 3 *(*PLD3*) gene segregates in two families with late-onset AD (LOAD) and doubles the risk of AD in European and African American cases/control samples [6], but this association failed to replicate in a subsequent study [7]. Likewise, a mutation in the* Triggering receptor expressed on myeloid cells 2 *(*TREM2*) gene was found to double the risk of AD in two independent case/control samples [8], associated in a family with frontotemporal lobar degeneration [9]. TREM2 is also overexpressed in brain tissue from individuals with AD [9].

Over the last 30 years, our group has studied the world's largest multigenerational pedigree in which a mutation in the* PSEN1* gene, also known as the* PSEN1* p.Glu280Ala E280A mutation (often referred to as the* Paisa* mutation), cosegregates with EOAD [2, 10]. This pedigree originated as a consequence of a founder effect [11] initially traced to 1783 [12] and localizes in a homogeneous environment [12, 13]. These two factors, along with the presence of exhaustive and detailed medical records of several hundred individuals, make this pedigree a powerful tool in genetic research [14–16]. Genome sequencing analysis successfully tracked the most common ancestor and the first mutation event for the E280 mutation to 10 and 15 generations ago, respectively [17].

To date, more than 5,000 individuals are descendants of the original founder, 1,784 of whom were enrolled to participate in comprehensive ongoing clinical monitoring. Of those, 459 mutation carriers and 722 noncarriers have been genotyped. Although the median age of onset (AOO) of AD in these individuals is ~49 years [95% CI 49-50], the broad spectrum of the AOO of dementia symptoms can be in the range of ~30–80 years [13, 16].

We previously identified both known and novel loci genome-wide significantly associated with AOO in AD, including* D-amino acid oxidase activator *(*DAOA*; rs778296, *P* = 1.58 × 10^−12^),* Homo sapiens CD44 molecule *(*CD44*; rs187116, *P* = 1.29 × 10^−12^),* Gremlin 2, DAN family BMP antagonist *(*GREM2*; rs12129547, *P* = 1.69 × 10^−13^),* Nephronophthisis 1 (juvenile) *(*NPHP1*; rs10173717, *P* = 1.74 × 10^−12^),* Homo sapiens Ca* ^*++*^ *-dependent secretion activator 2 *(*CADPS2*; rs3757536, *P* = 1.54 × 10^−10^),* Homo sapiens clusterin associated protein 1 *(*CLUAP1*; rs1134597, *P* = 1.12 × 10^−8^), and* Homo sapiens exocyst complex component 2 *(*EXOC2*; rs2804737, *P* = 3.28 × 10^−6^) [18]. Although the AOO modifier effect of the* NPHP1 *gene has been confirmed in a Caribbean population with AD and the G206A mutation in* PSEN1* [19], the functional assessment of the remaining variants was yet to be performed.

In this paper, we present the targeted analysis of functional exomic variants harboured in those genes reported as potential modifiers of the AOO of AD by a genome-wide association study (GWAS) [18]. We found that an exonic missense mutation in the* DAOA* (rs2391191, Arg30Lys, *P* = 1.94 × 10^−4^, *P*_FDR_ = 9.34 × 10^−3^) gene modifies the AOO in* PSEN1* E280A AD. Furthermore, nominal associations in* CLUAP1* (rs9790, Arg235Trp, *P* = 7.63 × 10^−3^, *P*_FDR_ = 0.1832) and* EXOC2* (rs17136239, Gln201Arg, *P* = 0.0325, *P*_FDR_ = 0.391) were also found. Clinical, biological, and mouse models evidence suggest that these functional coding variants are important players in shaping the susceptibility to AD, opening new windows towards outlining the genetic basis of this devastating neurodegenerative disease.

## 2. Methods

### 2.1. Subjects

**Figure 1:**
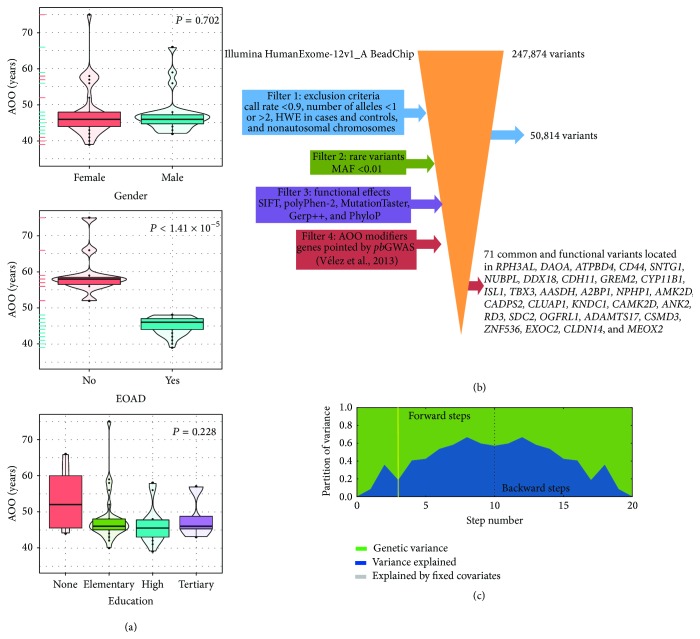
(a) Box- and violin-plots for the AOO of AD by gender (top), early-onset (middle), and level of education (bottom) in 60 patients carrying the* PSEN1* E280A  mutation. The associated *P* value after testing for differences in the average AOO is shown. AOO: age of onset; AD: Alzheimer's disease; EOAD: early-onset Alzheimer's disease. (b) Filtering workflow of exonic variants leading to the selection of 71 variants harboured in genes associated with modifiers of the AOO of AD in carriers of the* PSEN1* E280A mutation as reported by Vélez et al. [18]. Abbreviations as in (a). (c) Partition of phenotypic variance for each forward inclusion (steps 1 to 10) and backward elimination (10 steps after the dotted line). The yellow vertical line marks the model selected based on the lowest Bayesian Information Criterion (BIC).

Sixty patients with AD carrying the Paisa mutation, and displaying an extreme AOO, were selected from our clinical study for whole-exome genotyping (36 women [60%] and 24 men [40%]) [13]. The mean AOO of AD was 47.8 ± 6.4 years. No difference in the average AOO in AD was found by gender (female: 48.0 ± 7.02; male: 47.4 ± 5.52, *P* = 0.702) (Figure 1(a), top). A total of 49 patients (28 women [57%] and 21 men [43%]) had an AOO of AD below 48 years and* ad hoc* classified as EOAD, whilst the remaining 11 individuals were* ad hoc* classified as LOAD [18]. As intended, the average AOO was significantly different between EOAD and LOAD patients (EOAD: 45.1 ± 2.22, LOAD: 59.4 ± 6.15, *P* < 1.41 × 10^−5^) (Figure 1(a), middle). Years of education ranged from 0 to 19 years. Four patients (7%) never attended school, 30 (50%) completed primary school (grades 1 to 5), 22 (37%) completed high school (grades 6 to 11, inclusive), and only 4 (6%) had tertiary education. No difference was found in AOO of AD across education groups (*F*_3,56_ = 1.487, *P* = 0.228) (Figure 1(a), bottom).

### 2.2. Whole-Exome Genotyping

Genomic DNA from 60 participants was whole-exome genotyped by the Australian Genome Facility (Melbourne, VIC, Australia), an Illumina Certified Service Provider for the Infinium Genotyping Service. Briefly, DNA was whole-genome amplified, fragmented, hybridized, fluorescently tagged, and scanned [20]. Whole-exome genotyping was conducted using Illumina's HumanExome 12v1_A BeadChip. This chip covers regions with putative functional exonic variants selected from exome- and whole-genome sequences of >12,000 individuals. The exonic content consists of >250,000 markers representing diverse populations (including European, African, Chinese, and Hispanic individuals) in addition to common conditions (such as type 2 diabetes, cancer, and metabolic and psychiatric disorders). In order to test genotyping reliability and quality, one individual was duplicated. The identity by descent (IBD) matrix between all pairs of individuals was used for quality control and for subsequent analyses concerning the mixed model (see below). Entries of the IBD matrix contain the probability that a particular allele is inherited from a common ancestor [21].

### 2.3. Genetic/Statistical Analysis

#### 2.3.1. Quality Control and Filtering

Genotypes were extracted using the Genotyping module of Illumina's GenomeStudio v2010.3 (with the default settings) and the Illumina HumanExome 12v1_A manifest cluster file. Samples with calls below Illumina's expected 99% single nucleotide polymorphisms (SNPs) call rates were excluded. Genotype files were processed in Golden Helix SNP and Variation Suite (SVS) 8.0.2 (Golden Helix, Inc., Bozeman, MT, USA) using the GenomeStudio DSF Plugin. Golden Helix SVS is an integrated collection of analytic tools for managing, analyzing, and visualizing multifaceted genomic and phenotypic data.

For replication purposes, only variants located in the top 30 chromosomal regions reported as potential modifiers of the AOO in patients AD carrying the Paisa mutation [18] were included for further analysis. Marker exclusion criteria included (i) deviations from the Hardy-Weinberg equilibrium with *P* < 2 × 10^−7^ (0.05/250,000 markers) in both cases and controls (a stringent criterion to avoid the exclusion of any causal variant of major effect), (ii) a minimum genotype call rate of 90%, (iii) the presence of one or more than two alleles, and (iv) a MAF < 1%. Genotype and allelic frequencies were estimated by maximum likelihood.

#### 2.3.2. Filtering and Classification of Functional Variants

Exonic variants with potential functional effect were determined using the functional prediction information available in the dbNSFP_NS_Functional_Predictions GRCh_37 annotation track. This filter uses SIFT [22], PolyPhen-2 [23], MutationTaster [24], Gerp++ [25], and PhyloP [25] and is implemented in the SVS Variant Classification module. Variants were classified based on their potential effect on genes according to their position in a gene transcript, and those variants in coding exons were subsequently classified according to their potential effect on the gene's protein structure. This method gives insight into which variants are most likely to have functional effects.

#### 2.3.3. Genetic Analysis of Exonic Variants

Single- and multilocus additive linear mixed-effect models (LMEMs) [26–28] were fitted to test the association of these variants to AOO of AD. The advantage of these models is the inclusion of both fixed (sex and years of education) and random effects, the latter to account for kinship effects by including the IBD matrix. The single-locus LMEM assumes that all loci have a small effect on the trait, whilst multilocus LMEMs assume that several loci have a large effect on the trait. In a single-locus model, the association between the variant of interest and the disease trait is tested after covariates and genetic stratification are controlled for. Conversely, in a multilocus model the association is tested after covariates, genetic stratification, and the effect of the remaining *m* − 1 variants are controlled for. These recently emerging methods have been proven to be more powerful than existing methods [28]. Furthermore, this family of models allows handling of confounding effects and accounts for loci of small- and large-effect in structured populations with a small computational burden [28]. After the estimation process was finished, the coefficients ${\hat{\beta }}_{1},{\hat{\beta }}_{2},\ldots ,{\hat{\beta }}_{m}$ from the linear mixed-effects model were extracted and a hypothesis test of the form *H*_0,*i*_ : *β*_*i*_ = 0 versus *H*_1,*i*_ : *β*_*i*_ ≠ 0 was performed for the *i*th exonic variant to obtain the corresponding *P* value (*i* = 1,2,…, *m*). Thus, the collection *P*_1_, *P*_2_,…, *P*_*m*_ of *P* values was corrected for multiple testing using the false discovery rate (FDR) [29] and a method based on extreme-values theory [30]. Because the tests of hypothesis being performed are of the same type, correction was performed on the resulting *m* *P* values only [29, 30]. Exonic variants significantly associated with the AOO of AD were determined based on these derived *P* values.

## 3. Results

### 3.1. Quality Control

A total of 247,874 variants in the Illumina's HumanExome 12v1_A BeadChip were submitted to quality control. In the first filter, 50,814 variants with call rate < 0.9, in Hardy-Weinberg equilibrium in both cases and controls and located on autosomal chromosomes, were kept. This number was reduced to 71 common variants with potential functional effects at the end of the filtering process (Figure 1(b)). These resulting common variants are harboured in chromosomal regions reported as modifiers of the AOO in* PSEN1* p.Glu280Ala E280A AD, as reported by Vélez et al. [18].

### 3.2. Exonic Associated Variants

Multilocus additive LMEMs including all 71 common variants located in genes modifying the AOO in patients with* PSEN1* p.Glu280Ala E280A AD were fitted. Based on the Bayesian Information Criterion (BIC), a LMEM with three steps in the forward/backward selection algorithm [28] was selected (BIC = −50.8). In this model, the pseudoheritability (defined as the proportion of inheritance explained by the random effects) was 0.9987, whilst the proportion of genetic variance explained was ~20% (Figure 1(c), yellow vertical line). We found that variant rs2391191 (UCSC GRCh37/hg19 coordinates) is significantly associated with AOO in our sample of 60 individuals with AD carrying the Paisa mutation (*P* = 1.94 × 10^−4^, *P*_FDR_ = 9.34 × 10^−3^). Located in position 106,119,446 of chromosome 13, this is a missense variant (Arg30Lys) in the* DAOA* gene (NM_172370). Two more exonic variants were found to be nominally associated with the AOO in patients with* PSEN1* E280A AD: rs9790 (*P* = 7.63 × 10^−3^, *P*_FDR_ = 0.1832) mapping to chr 16: 3,586,230 (UCSC GRCh37/hg19 coordinates) and corresponding to a missense variant (Arg235Trp) in the* CLUAP1* gene (NM_015041) and rs17136239 (*P* = 0.0325, *P*_FDR_ = 0.391) which maps to chr 6: 656,343 (UCSC GRCh37/hg19 coordinates) and corresponds to a missense variant (Gln201Arg) in* EXOC2* (NM_018303).

## 4. Discussion

We previously reported that variants within or close to the* DAOA*,* CLUAP1*, and* EXOC2* genes were identified as AOO modifiers of AD in carriers of the* PSEN1 *E280A mutation [18]. Here, we report that a common functional exonic variant in* DAOA* modifies the AOO of AD in those patients. Although further studies are required to replicate this finding in other populations, this result suggests a potential genetic interaction [31] between* PSEN1* and* DAOA*, similar to what has been shown in genes involved in cholesterol, amyloid, inflammation, and oxidative stress in sporadic [32], late-onset [33], and familial AD [34].

The* DAOA* gene, also known as* G72*, is located in the 13q33.2 chromosomal region, spans 25,168 bp (UCSC GRCh37/hg19 coordinates), and its expression is enriched in the brain, spinal cord, and testis. In mice, G72 has been found to be overexpressed in testis and cerebral cortex, with low to no expression in other tissues [35].* DAOA*, which has typically been associated with bipolar disorder (BD) and schizophrenia (SZ), encodes a protein that may act as an activator of the DOA (*D-amino acid oxidase*) enzyme, which degrades the gliotransmitter D-serine, a potent activator of N-methyl-D-aspartate (NMDA) type glutamate receptors [36]. Polymorphisms in* DAOA *have been associated with the occurrence of neuropsychiatric symptoms such as depression, apathy, aggression, delusions, hallucinations, and psychosis in AD [37, 38]. In particular, the development of psychotic symptoms has been attributed to a similar psychosis-modifier gene mechanism to that in SZ because of the cytokine pathway disruption in both diseases [39, 40].

NMDA receptors (NMDARs) are glutamate-gated cation channels with high calcium permeability, critical for the development of the central nervous system (CNS), generation of rhythms for breathing and locomotion, and the processes underlying learning, memory, and neuroplasticity [41–44]. NMDARs regulate the functional and structural plasticity of individual synapses, dendrites, and neurons by activating specific calcium-dependent signaling cascades [44–46]. Specifically, both synaptic strengthening and weakening processes are mediated by Ca^2+^ influx through NMDARs [44]. Evidence in mouse models suggests that adult mice benefit from the genetic enhancement of the NMDAR function as it improves memory, but that blocking the NMDAR in the brain compromises learning and spatial memory as a consequence of the impairment of synaptic plasticity [41, 43, 46–49]. Furthermore, abnormal expression levels and altered NMDAR function have been implicated in numerous neurological disorders, including AD [44, 45], and therefore considered an important therapeutic target in this neurodegenerative disease [43–45]. In fact, a partial NMDAR antagonist, memantine, was approved to treat moderate to severe AD in the US and Europe [50, 51]. However, the success of memantine and other NMDARs has been limited in the clinical setting due to their low efficacy and side effects [44, 52, 53].

Two more exonic variants, one in* CLUAP1* (NM_015041) and one in* EXOC2* (NM_018303), were found to be nominally associated with the AOO in patients with the* PSEN1 *E280A AD. The* CLUAP1* gene spans 38,125 bp in the 16p13.3 chromosomal region and interacts with* APP*, the* Homo sapiens clusterin *(*CLU*),and the* Melanoma associated antigen 11 *(*MAGEA11*) genes [54]. Gene ontology analyses suggest an important role of* CLUAP1* in synaptic growth at neuromuscular junction, neuron remodelling, exocytosis, axon midline, and smooth endoplasmic reticulum calcium ion homeostasis. Mouse models support the role of Cluap1 in ciliogenesis due to the concentration of p75 neurotrophin receptors in the primary cilia membranes [55]. In humans, cilia are involved in numerous cellular activities [56] and have been suggested to impact cognitive deterioration in AD as a consequence of the neurogenesis process occurring in the hippocampus (which is necessary for new memory encoding) [57]. Subsequently, novel therapeutic approaches to AD, especially at the early stage of its development, have been outlined [57].

The* EXOC2* gene encodes a protein member of the exocyst complex. This complex, triggered in many ways by Ca^2+^ [58], is essential for tying exocytic vesicles to the plasma membrane [59]. In mice, higher total presynaptic mitochondrial volumes are associated with higher levels of exocytosis in stimulated hippocampal synaptosomes [47]. Furthermore, weighted gene coexpression analysis of posterior cingulate (PC) astrocytes in AD showed that* EXOC2* was part of the largest coexpressed modules, providing evidence that brain immunity and mitochondrial function in PC astrocytes are perturbed in AD [60]. These findings correlate with other studies suggesting an important role of astrocytes in AD, particularly in the earliest neuronal deficits [61], and their contribution to the neuroinflammatory component of neurodegeneration during latter stages of the disease [62].

## 5. Conclusions

Here we present a follow-up of our GWAS study linking several loci to the AOO of AD [18] in the world's largest genealogy segregating EOAD. Previous studies have linked polymorphisms in the* G72* (*DAOA*) occurrence of neuropsychiatric symptoms in AD, and this study confirms the existence of an AOO modifier mutation in the* DAOA* gene, a usual suspect associated with shaping the natural history of AD.
